# In Situ Gelling Ophthalmic Drug Delivery System for the Optimization of Diagnostic and Preoperative Mydriasis: In Vitro Drug Release, Cytotoxicity and Mydriasis Pharmacodynamics

**DOI:** 10.3390/pharmaceutics12040360

**Published:** 2020-04-15

**Authors:** Pierre-Louis Destruel, Ni Zeng, Françoise Brignole-Baudouin, Sophie Douat, Johanne Seguin, Elodie Olivier, Melody Dutot, Patrice Rat, Sophie Dufaÿ, Amélie Dufaÿ-Wojcicki, Marc Maury, Nathalie Mignet, Vincent Boudy

**Affiliations:** 1 Unither Développement Bordeaux, ZA Tech Espace, av Toussaint Catros, 33185 Le Haillan, France; pierre-louis.destruel@unither-pharma.com; 2 Département Recherche et Développement Pharmaceutique, Agence Générale des Equipements et Produits de Santé (AGEPS), AP-HP, 7 rue du fer à moulin, 75005 Paris, Francevincent.boudy@aphp.fr (V.B.); 3 Université de Paris, UTCBS, CNRS, INSERM, Faculté de Pharmacie, 4 av de l’observatoire, 75006 Paris, France; 4 Unither Pharmaceuticals, 3-5 rue St-Georges, 75009 Paris, France; 5 UMR CNRS 8038-Chimie Toxicologie Analytique et Cellulaire, 75006 Paris, France; 6 CNRS UMR 7210-Inserm UMR_S 968, Institut de la Vision, 75012 Paris, France; 7 Centre Hospitalier National d’Ophtalmologie des Quinze-Vingts, INSERM-DHOS, CIC 503, 75012 Paris, France; 8 Recherche et Développement, Laboratoire d’Evaluation Physiologique, Yslab, 2 rue Félix le Dantec, 29000 Quimper, France

**Keywords:** mydriasis, drug release, in situ gelling, ophthalmic, delivery system, gellan gum

## Abstract

Mydriasis is required prior to many eye examinations and ophthalmic surgeries. Nowadays, phenylephrine hydrochloride (PHE) and tropicamide (TPC) are extensively used to induce mydriasis. Several pharmaceutic dosage forms of these two active ingredients have been described. However, no optimal therapeutic strategy has reached the market. The present work focuses on the formulation and evaluation of a mucoadhesive ion-activated in situ gelling delivery system based on gellan gum and hydroxyethylcellulose (HEC) for the delivery of phenylephrine and tropicamide. First, in vitro drug release was studied to assess appropriate sustained drug delivery on the ocular surface region. Drug release mechanisms were explored and explained using mathematical modeling. Then, in situ gelling delivery systems were visualized using scanning electron microscopy illustrating the drug release phenomena involved. Afterward, cytotoxicity of the developed formulations was studied and compared with those of commercially available eye drops. Human epithelial corneal cells were used. Finally, mydriasis intensity and kinetic was investigated in vivo. Mydriasis pharmacodynamics was studied by non-invasive optical imaging on vigilant rabbits, allowing eye blinking and nasolacrimal drainage to occur physiologically. In situ gelling delivery systems mydriasis profiles exhibited a significant increase of intensity and duration compared with those of conventional eye drops. Efficient mydriasis was achieved following the administration of a single drop of in situ gel reducing the required amount of administered active ingredients by four- to eight-fold compared with classic eye drop regimen.

## 1. Introduction

Prior to a great number of eye examinations or ophthalmic surgeries, dilation of the pupil is required. Association of tropicamide, a muscarinic antagonist, and phenylephrine, an adrenergic receptor agonist, provides efficient pupil dilation and long duration of action, both therapeutic classes acting synergistically [1]. Nowadays, different therapeutic strategies are available and used routinely to induce pupillary dilation, also called mydriasis [2]. 

Mydriasis is a physiological phenomenon controlled by the iris, composed of two antagonistic muscles controlling its opening, and allowing the eye to adapt to the surrounding light intensity. When there is little light, the pupil dilates under the action of the radial dilator smooth muscle, this is the mydriasis. Conversely, when the light intensity is high, the pupil contracts under the action of the circular sphincter muscle, this is the miosis. The diameter of the pupil can vary from 0.5 to 13 mm. Physiologically, these two phenomena are governed by the autonomic nervous system [3]. The radial dilator muscle is innervated by the sympathetic system, whereas the sphincter muscle is innervated by the parasympathetic system [4].

To achieve therapeutic mydriasis, the main strategy consists in the association of two types of liquid eye drops (tropicamide 0.5% and phenylephrine 2.5%, 5%, or 10%). However, the administration of drugs at the ocular surface for local action by conventional eye drops often leads to low ocular bioavailability (5%–10%) because of eye blinking and nasolacrimal drainage [5], resulting in high absorption at the systemic level (50%–90%) and potentially leading to severe side effects [6,7,8]. Furthermore, the rapid elimination of the administered eye drops often results in a short duration of the therapeutic effect, therefore requiring frequent administrations. To achieve sufficient mydriasis, three to five drops of each eye drop with a five minutes interval between each drop are required to obtain an effective mydriasis within 30 to 45 min.

The second strategy is based on the administration of a solid insert of phenylephrine and tropicamide (Mydriasert^®^) [9]. The insert is an osmotic tablet, placed in the lower conjunctival bag by a trained medical staff and must remain for 30 to 45 min to induce a sufficient mydriasis, causing some discomfort for the patient [10]. 

Recently, a third strategy consisting of an intracameral injection of tropicamide, phenylephrine, and xylocaine (Mydrane^®^) was brought to the market. This product is only indicated for pre-surgery mydriasis because of the presence of an anesthetic in its composition and its administration route [11,12].

To overcome the drawbacks of existing dosage forms and meet an important need of clinicians and patients, a novel mydriatic in situ gelling delivery system was developed [13]. Many investigators have considered the use of in situ gelling delivery systems as a promising way to achieve efficient topical ophthalmic drug delivery [14]. As a result of their physicochemical properties, in situ gelling systems allow an easy, safe and reproducible administration as a liquid drop and exhibit a sol to gel transition onto the ocular surface. Most often, this phase transition is observed for stimuli responsive polymeric formulations under variations of pH, temperature or ionic environment [15]. On the one hand, the liquid phase allows the formulation to spread over a larger area than solid forms, resulting in a larger absorption surface. On the other hand, the gel phase allows prolonged residence time of the formulation on the ocular surface. Thus, in situ gelling systems exhibit the main requirements of a topical ophthalmic dosage form. Hence, suitable mechanical and mucoadhesive properties along with appropriate release properties, and extended residence time on the ocular surface should be exhibited [16].

In a previous work [13], three formulations of in situ gelling delivery systems were developed and characterized rheologically. Their extended retention time was then assessed in vivo. Herein, in vitro release studies were conducted to investigate the kinetic of drug release and better comprehend the different release mechanisms involved. The prolonged delivery of the drugs was assessed. 

Then, cytotoxicity of the hydrogels was investigated in vitro. As ophthalmic products, the in situ gelling delivery systems should be harmless for administration on the ocular mucosa. Toxicities of developed in situ gelling delivery systems and commercially available eye drops were compared on human epithelial corneal cells (HCE). 

Finally, the efficacy and pharmacologic effects were assessed in vivo. Mydriasis intensity was recorded by optical imaging on New Zealand albino rabbits. Again, the mydriatic effect of in situ gelling delivery systems was compared with commercially available eye drops.

## 2. Materials and Methods 

### 2.1. Materials

All the hydrogels were prepared using sterile water Versylene^®^ purchased from Fresenius Kabi France (Sèvres, France). Phenylephrine hydrochloride (PHE) of European Pharmacopoeia grade was a kind gift from Cheng Fong Chemical Co., Ltd. (Taipei, Taiwan). Tropicamide (TPC) of European Pharmacopoeia grade was a free sample from Tokyo Chemical Industry Co., Ltd. (Tokyo, Japan). Deacylated gellan gum (Kelcogel^®^ CG-LA) of pharmaceutical use grade was a free sample from CP Kelco (Atlanta, GA, USA). Hydroxyethylcellulose (HEC) (Natrosol^®^ 250 M) was generously provided by Ashland (Schaffhausen, Switzerland). Sodium citrate was purchased from Cooper (Ponthierry, France).

### 2.2. In Situ Gelling Formulations

The hydrogels were prepared as in our previous work [13]. Final concentrations are reminded in Table 1.

### 2.3. High Performance Liquid Chromatography (HPLC) Analysis of Phenylephrine Hydrochloride and Tropicamide

The HPLC system consisted of a Dionex UltiMate 3000 Series equipped with a quaternary pump, online degasser, autosampler, and photodiode array UV/vis detector. Data collection and analysis were performed using Chromeleon 7.6.2. (Dionex, France). The current method was adapted from literature and validated as per ICH Q2R1 guideline. Briefly, the separation was achieved on a Kinetex C18 column (5 µm, 4.6 mm × 250 mm). The elution was isocratic at 1.0 mL/min with a mobile phase of 72% (*v*/*v*) octane sulfonic acid buffer (pH 3.5) and 28% (*v*/*v*) acetonitrile. The injection volume was 20 µL and UV detection was at 254 nm for TPC and 272 nm for PHE. Linearity was established in the concentration range of 8–120 µg/mL (r^2^ > 0.999) for PHE and of 0.8–12 µg/mL (r^2^ > 0.999) for TPC, and the method was precise (<2.5% relative standard deviation for both intra- and inter-day variation) and accurate (>98.5% recovery). The limit of detection (LOD) and limit of quantitation (LOQ) were determined to be 0.17 µg/mL and 0.5 µg/mL for PHE and 0.27 and 0.8 µg/mL for TPC, respectively. The standard curve, constructed as described above, was used for determining PHE and TPC quantities.

### 2.4. In Vitro Drug Release Study Using the USP 4 Apparatus

Various in vitro release studies of ophthalmic semisolid dosage forms were carried out using different apparatus [17]. Classic in vitro release studies focus only on the diffusion of the drug out of its dosage forms, often with an intervening membrane (e.g., dialysis methods, Franz diffusion cells). It was shown that those methods cannot be considered biorelevant regarding topical administration as different efflux mechanisms such as eye blinking and nasolacrimal drainage have a significant impact on the release mechanisms involved. 

The study of erosion phenomenon using a flow through apparatus showed promising results in the prediction of in vivo retention time [18]. Flow through apparatus is now extensively used for in vitro drug release tests from ophthalmic dosage forms allowing the testing under pseudo-physiological conditions in term of flow rate and release medium ratio [19]. 

All the drug release experiments were carried out using a USP 4 apparatus (flow through cell) [20] with two cell models: the standard cell without membrane and an adapter for semisolid forms with membrane. This investigation was initiated to study the in vitro release profiles of formulations A, B, and C, and therefore, the influence of HEC on the release mechanisms. 

Simulated tear fluid (STF) (pH 7.4) was used as the release media. Phenylephrine hydrochloride (PHE) is a class I molecule in the BCS classification, its solubility in aqueous media is considered high, providing “sink conditions” in our in vitro release models. Contrariwise, Tropicamide (TPC) is a class II molecule in the BCS classification, its solubility in aqueous media is then considered low. However, the low quantity of TPC compared with the relatively high volume of release media provided sink conditions as well.

#### 2.4.1. In Vitro Drug Release Evaluation Using the Standard Flow-Through Cells

To study the release properties and differentiate the three formulations, six standard flow-through cells with a diameter of 22.6 mm were used in all experiments. Operated in the closed configuration, the automated system CE7 smart Sotax (Basel, Switzerland) was linked to an Ismatec MCP peristaltic pump (Wertheim, Germany). Release media were sampled manually and further analyzed using the HPLC system described above. In each cell, a ruby bead of 5 mm in diameter and glass beads of 1 mm in diameter were placed in the apex of the flow-through cells in order to ensure laminar flow. Approximately 300 µL of hydrogel were placed into the glass bead bed. During the test, 150 mL of STF were pumped through each cell with flow rates of 3, 8, and 15 mL/min. Temperature of 35 ± 0.5 °C, i.e., temperature of the ocular surface, was maintained throughout the study. Aqueous solution of PHE 5% and TPC 0.5% was also tested and used as reference. The results are the mean ± SD of n = 6 experiments. Drug release profiles obtained with the closed configuration, i.e., cumulative percentage of drug release (*M_t_*/*M*_1_, %) versus time (*t*, min), were plotted.

#### 2.4.2. In Vitro Drug Release Evaluation Using the Semisolid Adapter

To evaluate the diffusion profiles of PHE and TPC from the hydrogels, an adapter for semisolid dosage forms [21] was used. The adapter was designed to be used with the 22.6 mm flow-through cell of USP 4 apparatus. In brief, the adapter consists of two basic components: a reservoir where the product is introduced and a ring where the membrane is held. In total, 1200 µL of hydrogel was placed in the reservoir, and the ring, once fitted with an acetate cellulose membrane (0.45 µm), was screwed onto the reservoir with a specific tool. The adapter was then slid into the cylindrical part of the cell with the membrane facing downward. A closed system was applied as well. The flow rate was set at 15 mL/min. The testing procedure was the same as previously described in the standard cell. 

### 2.5. Mathematic Modeling of Drug Release Kinetics

To investigate the kinetics of drug release from hydrogels, several mathematical models were applied [22]. A combination of theoretical, semi-empirical, and empirical models was used to fully understand the release mechanisms involved in the release of phenylephrine and tropicamide from the in situ gels. Cumulative amounts of drug release (%) as a function of time were fitted to Higuchi [23], Korsmeyer-Peppas [24], and Peppas-Sahlin [25] models, along with Weibull [26,27], Hopfenberg [28], and Makoïd-Banakar models. The adjusted coefficient of determination (R^2^_adjusted_) was used as a meaningful criterion for the selection of the “best” models [29]. The complex and unpredictable shape of the gel samples in the glass beads bed did not allow an accurate use of shape dependent models such as Higuchi and Korsmeyer-Peppas. The Peppas-Sahlin model, which can be used to analyze the first 60% of a release curve regardless of the geometric shape and theoretical models not originally based on in vitro release data were used. Thus, the Peppas-Sahlin, Makoïd-Banakar, Weibull and Hopfenberg models were applied to our data. 

Equations and descriptions of the models are detailed in Table 2. All the calculations were performed using DDSolver [30].

The drug release from semi-solid dosage forms is a complex phenomenon. Two major mechanisms are generally involved. The release can be either governed by Fickian diffusion (Case-I) or by relaxation/erosion of the system (Case-II transport) [32]. In many cases, both mechanisms are involved, and the release is therefore called anomalous.

Previous work from our group already emphasized the use of the Peppas-Sahlin model to quantify the contribution of diffusion and erosion in the drug release of a thermosensitive in situ gelling delivery system [18,31]. The Peppas-Sahlin equation can be transformed. The percentages of diffusion (Equation (1)) and percentages of erosion (Equation (2)) at time *t* were obtained by Zeng’s equations, assuming a *m* value of 0.5 regarding the geometry of the system:

(1) $$\frac{M_{t1}}{M_{t}}=\frac{1}{1+(k_{2}/k_{1})t^{1/2}}\times 100,$$

(2) $$\frac{M_{t2}}{M_{t}}=100-\left(\frac{1}{1+(k_{2}/k_{1})t^{1/2}}\times 100\right),$$

Similarity factor f2, a model-independent method, was calculated for the pair-wise comparison of all the release profiles. First, a comparison of release performances between PHE and TPC was done. Then, comparisons between formulations A, B, and C were done at each flow rate. 

Additionally, times for 25%, 50%, and 80% release (T_25_, T_50_, and T_80_, respectively) were calculated from the Peppas-Sahlin equation. 

### 2.6. Polymer Network Microstructure

The gel network microstructures were visualized using scanning electron microscopy (SEM). Samples were freeze-dried and mounted on specimen stubs, sputter coated with palladium using JEOL JCF100 and examined at 10 kV using JEOL ISM-35CF. Blank formulations, i.e., A and C without PHE and TPC, were observed before and after addition of STF. The impact of HEC on the arrangement and tortuosity of the polymer network was observed as well as the polymer entanglement due to the presence of mono- and divalent cations.

### 2.7. Cytotoxicity Assay on Human Corneal Epithelial Cells

#### 2.7.1. Cell Culture

Human corneal epithelial cells (HCE-T cell line, RCB-1384; Riken Cell Bank, Tsukuba, Japan) were cultured in Dulbecco’s Modified Eagle Medium: Nutrient Mixture F-12 (DMEM/F12, Gibco, Grand Island, NY, USA) supplemented with 10% fetal bovine serum (Gibco), 1% glutamine (Gibco), and 0.5% penicillin and streptomycin (Gibco). Cultures were maintained at 37 °C in 5% CO_2_ in a humidified incubator.

#### 2.7.2. Cell Incubation

When the cells reached confluence, they were detached from the flask with trypsin, centrifuged, and seeded in 96-well plates at 8 × 10^4^ cell/mL (200 µL per well) for 24 h prior to exposure to agents. The cells were then rinsed with PBS and incubated for 30 min with 100 μL of in situ gels. Blank gels (i.e., formulations A, B, and C without active ingredients), control solution of PHE 5% and TPC 0.5%, control solution of NaCl 1.29% (i.e., exhibiting the same osmolarity than the in situ gels) and commercially available eye drops (sterile unit doses of Mydriaticum^®^ 0.5% and Néosynephrine^®^ 10%) were used as controls. Cells were then washed with PBS. Neutral red uptake assay was performed immediately after the incubation and after a 24-h recovery time in the culture medium. Experiments were performed in triplicate with a minimum of six wells per condition.

#### 2.7.3. Cell Viability

Cell membrane integrity was evaluated using Neutral Red dye. The Neutral Red solution at 0.4% in water was diluted in cell culture medium with a ratio of 1:79 to give a final concentration of 50 μg/mL. Neutral Red was distributed in the plates for a three-hour incubation time at 37 °C. The cells were then rinsed with PBS to remove any remaining unincorporated dye. The dye was then released from the cells using a lysis solution (1% acetic acid, 50% ethanol, and 49% H_2_O) and the fluorescence was measured (λex = 540 nm, λem = 600 nm) using a microplate fluorometer (Spark, Tecan, Lyon, France).

### 2.8. In Vivo Evaluation of the Mydriasis

This animal research project was authorized by Ministry of higher education and research, in conformity with the regulations of Committee on Ethics in the care and use of laboratory animals (Comité d’Ethique pour l’expérimentation animale de Paris Descartes (CEEA 34)), with the project reference Apafis #14792, approved on 15 May 2018. Male New Zealand albino rabbits were purchased from CEGAV (France). The in vivo experiments were performed in non-anesthetized rabbits kept in restraining boxes. However, their heads were free of movements, so that normal eye blinking, head, and eye movements were allowed during the experiments.

One drop of preparation was carefully administered into animal’s conjunctival cul-de-sac with a plastic transfer pipette. The contralateral eye was treated with saline solution and used as control. The volume of the drops from different formulations as well as control solution was measured, and no significant variation was found (from 28.6 ± 0.8 μL to 30.1 ± 1.2 µL). A flexible scale was placed on the rabbit cheek [33], and pictures of both eyes were recorded using a Canon EOS 350D equipped with a Canon EF 100 mm f/2.8 Macro lens providing a zoom and an autofocus enabling to capture with a very high sharpness the entire ocular region and then to focus on regions of interest. Rabbits were placed under surgical light (Halux LED 20 P SX, Derungs medical lighting) to maintain constant lighting of the eye and avoid physiological variation of the mydriases. Photos of the rabbit’s eyes were taken before administration (reference image) and until five hours after formulation administration. 

Formulations A, B, and C were compared with a control solution (CTRL) (phenylephrine 5% and tropicamide 0.5%, in sterile water) and to commercially available eye drops (CED): Mydryaticum^®^ (tropicamide 0.5%) and Néosynéphrine^®^ (phenylephrine hydrochloride 10%). In the case of commercial eye drops, a standard protocol used clinically was followed: four drops of each eye drop with a five-minute interval between each drop were instilled from *t*_0_ to *t*_35__min_. The kinetic data of mydriasis were finally obtained by analyzing the images by open source processing program Image J [34]. The flexible scale placed on the cheeks of the Rabbits allowed rescaling the photos to avoid distance bias between the objective of the camera and the head of the Rabbits that were free of movements. Diameters of the pupil were measured, and diameters minus reference diameter were plotted against time to express the dilatation of the pupil. Area under curve (AUC) was calculated. Results are mean ± SD of *n* = 3 experiments, made on three different rabbits. Each rabbit had a minimum of 48 h washout period between two experiments.

At the end of the protocol, rabbits were placed in foster care by an approved association; no sacrifice was needed. 

### 2.9. Statistical Analysis

The statistical significance of the obtained values was analyzed using one-way ANOVA and multiple T-tests (*p* < 0.05) (Statgraphics centurion 18, Statgraphics Technologies, inc., The plains, VA, USA). For the comparison of different drug release profiles, similarity factor f2 was calculated using the DDsolver program. As the factor is sensitive to the number of time points, a limit of data (one time point after 60% drug release for the models and one time point after 85% drug released for the similarity factor) was analyzed. Two profiles were considered similar if f2 values were included between 50 and 100.

## 3. Results and Discussion

In a previous work [13], the physico-chemical, rheological and mucoadhesive properties of the in situ gelling delivery systems were characterized. The three formulations exhibited suitable pH (6.4 to 6.6), osmolality (429 to 443 mOsm/kg), and transmittance (>90%) for ophthalmic use. Viscosities and in situ gelation capacities were shown to be favorable to ocular administration. The addition of HEC enhanced the viscosity while decreasing the gels resistance to shear stress. HEC also reinforced the mucoadhesive properties of the formulations. Finally, ocular residence time was assessed in vivo, and the three formulations exhibited prolonged retention time (>3 h) on the ocular surface.

### 3.1. In Vitro Drug Release

#### 3.1.1. In Vitro Drug Release Evaluation Using the Standard Flow-Through Cells

**Figure 1:**
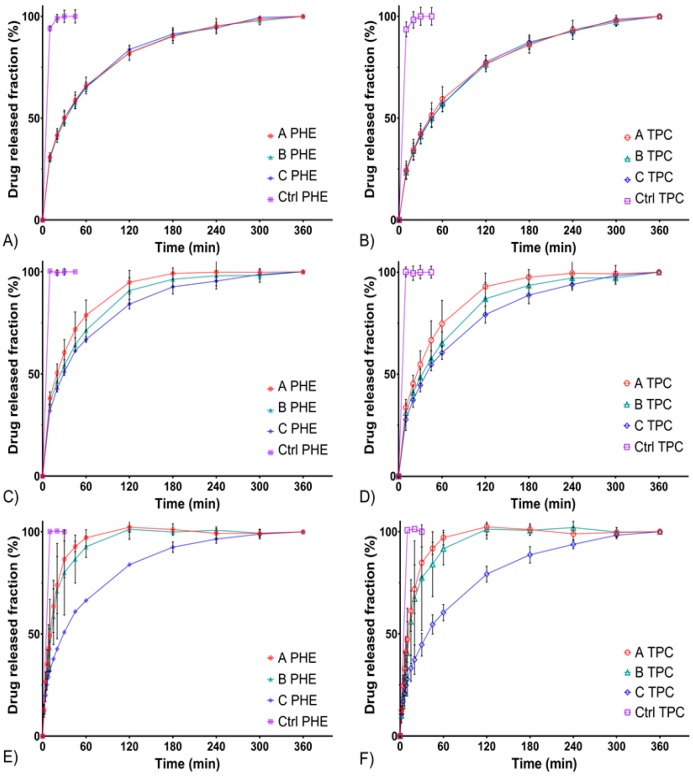
In vitro drug release profiles from formulations A, B, and C compared with a liquid control solution using the USP 4 standard flow through cells. PHE release profiles at 3 mL/min (**A**), 8 mL/min (**C**), and 15 mL/min (**E**) flow rates. Tropicamide (TPC) release profiles at 3 mL/min (**B**), 8 mL/min (**D**), and 15 mL/min (**F**) flow rates.

Drug release from semi-solid dosage forms is complex, various mechanisms are involved (i.e., diffusion and erosion) and numerous evaluation methods have been described and developed to study these parameters [19,35]. The results below showed that in standard flow-through cells, on an FDA-approved USP 4 apparatus, in situ gelling delivery systems exhibited a fast but prolonged release over time (Figure 1). All control solutions (CTRL) released more than 80% of the drugs in less than 10 min, whereas the 80% release time (T_80_) of formulations A, B, and C were reached between 30 min and 180 min depending on the flow rate. The prolonged drug release over time can be explained by two mechanisms. Either the release of the drug from the matrix is due to diffusion, or release of the drug is a result of erosion of this matrix. A slight initial burst release was observed, which turned out to be an asset regarding the rapid induction of mydriasis expected after administration [36].

To compare release profiles, a model-independent approach is recommended by the FDA. It involves the study of the similarity factor f2, which makes it possible to compare profiles easily [37]. A value of 100 for f2 factor means that the two profiles compared are completely identical and a value of 50 marks a difference of 10% over all time points. Thus, two profiles are considered similar (i.e., less than 10% of difference) if the f2 value is between 50 and 100. f2 values obtained comparing release profiles of PHE and TPC from formulations A, B, and C at three different flow rates are summarized in Table 3. 

Firstly, the release profiles of PHE and TPC were similar within each formulation at all flow rates (for all “PHE; TPC” profiles, f2 values were included between 50 and 100). At a flow rate of 3 mL/min, release profiles of formulations A, B, and C were similar. Then, at a flow rate of 8 mL/min, formulations A and C were not similar (f2 = 48.9 and 49.4 for PHE and TPC, respectively). Finally, at a flow rate of 15 mL/min, formulations A and B were not similar to formulation C. A low flow rate of 3 mL/min did not allow differentiating formulations A, B, and C. However, increasing the flow rate allowed highlighting the differences between the formulations.

Lastly, the release profiles of formulation C were similar regardless of the flow rate (Table 4). These differences at higher flow rates could be due to a higher sensitivity to erosion of formulations A and B compared with formulation C. 

To better comprehend the release mechanisms occurring and leading to these differences, a model-dependent approach was considered [38]. Results of fit (R^2^_adjusted_) and values of relevant parameters described in Table 2 are summarized in Table 5.

Makoïd-Banakar and Weibull models exhibited the best R^2^_adjusted_ at all flow rates (Table 5). However, the other applied models presented a relatively high goodness of fit. 

**Figure 2:**
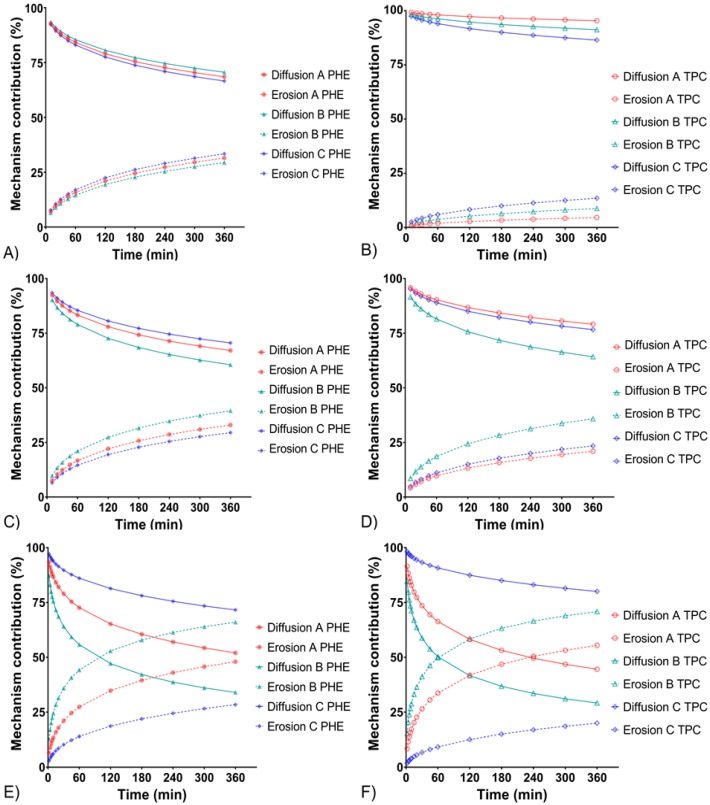
Percentages of diffusion and erosion contributions as a function of time of formulations A, B, and C. PHE mechanism contributions at 3 mL/min (**A**), 8 mL/min (**C**), and 15 mL/min (**E**) flow rates. TPC mechanism contributions at 3 mL/min (**B**), 8 mL/min (**D**) and 15 mL/min (**F**) flow rates.

When applying the Peppas-Sahlin model, two constants are obtained: *k_1_* and *k_2_*, which are, respectively, the diffusion and erosion constants. From *k_1_* and *k_2_* values, diffusion and erosion contribution percentages were obtained from Equations (1) and (2), respectively. Diffusion and erosion contributions were plotted against time in Figure 2.

The diffusion mechanism was predominant throughout the experiments for all formulations at 3 and 8 mL/min flow rates and formulation C at 15 mL/min. Their release mechanisms were similar (about 70% diffusion and less than 30% erosion). Regarding formulations A and B at a flow rate of 15 mL/min, erosion phenomenon increased. A crossover in the diffusion and erosion contribution curves was observed as erosion became predominant. Thus, the presence of a high concentration of HEC would allow formulation C to better withstand erosion phenomenon, confirming observations made from other models [36].

The times for 25%, 50%, and 80% of total released amount were also calculated from the Peppas-Sahlin model equation and the average values are shown in Table 6. For all formulations, the increase of the flow rate resulted in the decrease of T_25_, T_50_, and T_80_. Looking more closely at the T_80_ of formulations A and B, the drug release was much faster at a flow rate of 15 mL/min than at 3 mL/min (T_80_ decreased by four-fold). For formulation C, T_80_ were in similar range at a flow rate of 15 mL/min compared to 3 mL/min. Thus, the higher amount of HEC contained in formulation C would better maintain a sustained release over time, despite the increase of the flow rate. 

In Table 5, considering the Hopfenberg model, which reflects erosion, R^2^_adjusted_ values were close to 1 (>0.99) for formulations A and B at a flow rate of 15 mL/min. These results indicated, for these formulations and condition, a drug release mainly governed by erosion. In contrast, formulations A and B at 3 and 8 mL/min flow rates as well as formulation C at all tested flow rates, exhibited lower R^2^_adjusted_ values (<0.95), and thus, the diffusion phenomenon would have a greater role on the drug release.

The use of complementary theoretical models has been extensively described in the literature. Originally, the Weibull function and the Makoïd-Banakar model were not developed from any kinetic or drug release data. However, many investigators considered their use to investigate drug release profiles and demonstrated the importance of these models to provide additional information to those obtained by conventional drug release models [29]. Indeed, the goodness’s of fit of these two models were the best for all formulations with R^2^_adjusted_ > 0.998 (Table 5). The shape parameter *β*, from the Weibull model, confirmed that the release of PHE and TPC were governed by Fickian diffusion (*β* < 0.75) for all formulations except A and B at 15 mL/min flow rate, which were subject to the erosion of their matrix at this flow rate (*β* > 0.95). As the *C* parameter of the Makoïd-Banakar model was almost zero, the release exponent *n* became similar to the release exponent of the Korsmeyer-Peppas equation (Table 2). Herein, the *n* values of the Makoïd-Banakar model were <0.45, for all formulations at 3 and 8 mL/min flow rates, showing that diffusion was the principal mechanism involved in the release of PHE and TPC [27]. These findings still excluded both formulations A and B at a flow rate of 15 mL/min, for which *n* values were >0.85 and indicated their sensitivity to erosion in this condition.

#### 3.1.2. In Vitro Drug Release Evaluation Using the Semisolid Adapter

Drug release on the ocular surface is complex to simulate as efflux mechanisms of a different nature occur (i.e., tear fluid dilution or destructive shear stress during eye blinking). Moreover, these phenomena exhibit an important interpersonal variability. Therefore, conventional release testing methods do not allow an accurate measurement of the amount of drug released in vivo on the ocular surface region [18,39]. To prevent the drug release related to erosion and compare, from an unbiased point of view, the relatively absolute diffusion profiles of formulations A, B, and C, the adapter for semisolid forms was also used in our work. The release parameters of the in situ gels are shown in Table 7.

**Figure 3:**
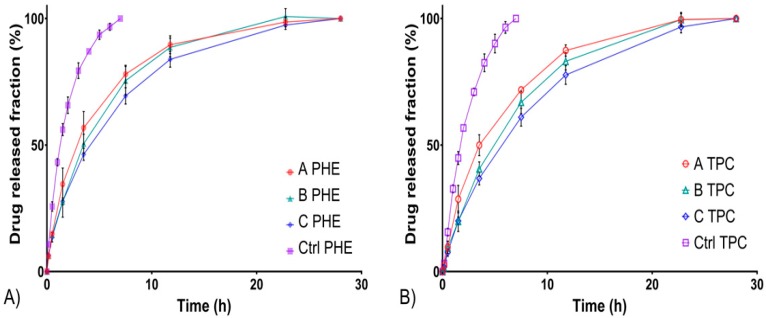
In vitro drug release profiles from formulations A, B, and C compared with a liquid control solution using the semi-solid adapter. PHE (**A**) and TPC (**B**) release profiles at 15 mL/min flow rate.

The results below showed that in flow-through cells equipped with semi-solid adapter (Figure 3), in situ gels had the ability to maintain a prolonged release for more than 20 h. In this experiment, the release rate is impacted by two components: the release of the APIs from the gel and the diffusion of the APIs through the semi-permeable membrane. T_80_ of formulations A, B, and C, were reached between 7.5 and 16 h. Additionally, as already observed using the standard cells, a slight burst release occurs at initial times.

In comparison, in the case of the control solution, all the APIs in solution are readily available and the release rate can be related to the diffusion of the APIs trough the semi-permeable membrane only. Here, 80% of the initial amount of PHE and TPC is released in about 4 h for control solutions (CTRL) exhibiting the delay due the diffusion through the semi-permeable membrane. This delay must be taken into account; however the in situ gels allowed a significant extended release compared to the solution. 

From Table 7 and Figure 3, a trend could be pointed out: the release of PHE and TPC from formulation A was faster than that of formulation B, itself faster than that of formulation C. This was consistent with drug release profiles obtained using the standard cells. Thus, in addition to improving erosion resistance, HEC also extended the release due to diffusion. 

### 3.2. Polymer Network Microstructure

**Figure 4:**
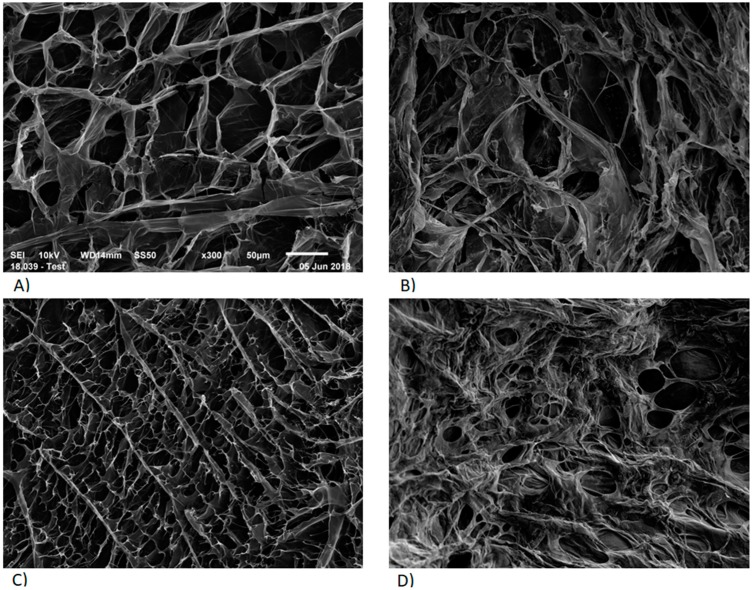
SEM micrographs of the internal structure of freeze-dried in situ gelling systems. Formulation A without PHE and TPC, before (**A**) and after addition of STF (**B**). Formulation C without PHE and TPC, before (**C**) and after addition of STF (**D**).

To better understand and explain the previous observations on in vitro drug release, the microstructures of the in situ gels networks were visualized by SEM. It can be observed that the polymer networks of formulations A (Figure 4A) and C (Figure 4C) before addition of STF were quite organized. As expected, the network structure of formulation C being denser due to the presence of HEC. After addition of STF, formulations A (Figure 4B) and C (Figure 4D) revealed less porous spiderweb-like polymer networks, similar as the observations made by Rupenthal et al. [16] and Fernandez-Ferreiro et al. [40]. 

In the first part of this work, the effect of the addition of STF on gelation was assessed rheologically. The complete change in polymer arrangement and entanglement due to the addition of STF agreeing with the gelation mechanism of gellan gum [41]. The presence of leaflets, smaller lamellar distances, and shorter mesh was consistent with the polymer chains cross-linking as a result of ionic interactions with mono- and divalent cations contained in the STF. Therefore, these micrographs confirmed the in situ gelling character of the developed formulations.

With reference to in vitro drug release observations, the tighter pores observed in the presence of HEC (Figure 4D) were likely to prolong the diffusion of the drug. Indeed, increasing the tortuosity of hydrogels has demonstrated to influence the drug release characteristics by limiting the possible routes for the drug to diffuse out of the matrix [42,43]. Moreover, the denser structure could explain an increased resistance to water uptake and erosion observed at high flow rates, highlighting the impact of HEC on the prolonged drug delivery of in situ gelling systems. 

### 3.3. Cytotoxicity Assay on Human Corneal Epithelial Cells

**Figure 5:**
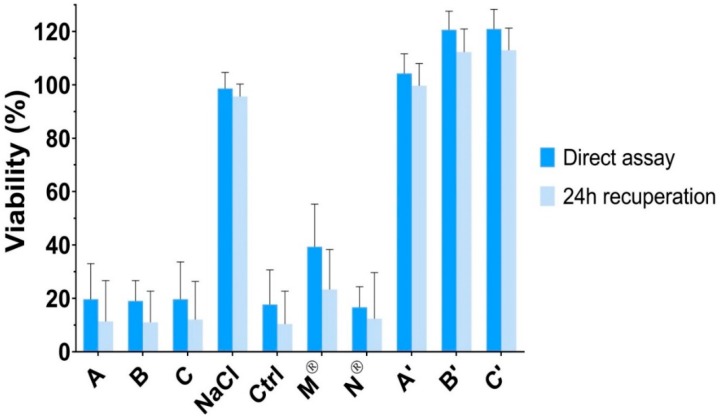
Neutral red cell viability assessment after exposure to formulations A, B, and C, NaCl 1.29% osmotic control solution, liquid control solution of PHE 5% and TPC 0.5%, commercially available eye drops (Mydriaticum^®^ 0.5% and Neosynephrine^®^ 10%) and to formulations A, B, and C without active ingredients (respectively A’, B’, and C’). The viability was calculated using untreated cells as reference.

The results of the cytotoxicity assay are presented in Figure 5. First, formulations A, B, and C induced a significant reduction of viability when applied on HCE cells (*p* < 0.001), with no recuperation after 24 h. For the control solution of NaCl 1.29%, which presented an osmolarity value of 430 mOsm/L, no impact on cell viability was observed (viability of 98.7 ± 6.0%). Therefore, the relatively high osmolarity of formulations A, B, and C was not considered as the parameter inducing cytotoxicity.

Then, a control solution containing PHE 5% and TPC 0.5%, was tested. A significant decrease of viability was found (*p* < 0.001), which was similar to those observed for formulations A, B, and C (*p* > 0.05). In addition, commercially available eye drops of PHE (Néosynéphrine^®^ 10%) and TPC (Mydriaticum^®^ 0.5%) were tested individually. Again, Néosynéphrine^®^ 10% induced a significant reduction of viability with similar values than formulations A, B, and C (*p* > 0.05). Mydriaticum^®^ 0.5% induced a lower diminution of the cell viability, but still, viability was not higher than 39.3 ± 16.1%. Hence, the apparent cytotoxicity exhibited by formulations A, B, and C should be due to the cytotoxicity induced mainly by PHE but also by TPC. 

Finally, corneal epithelial cells membrane integrity was not altered by the in situ gels formulations without PHE and TPC (formulations A’, B’, and C’) and remained above 100%. Neutral red assay indicated no significant difference in viability after exposure to formulations A’, B’, and C’ and untreated cells (*p* > 0.05). Accordingly, the developed delivery systems did not induce cytotoxicity, which was due to the contained active ingredients. Nevertheless, PHE (2.5% to 10% eye drops) and TPC (0.5% to 1% eye drops) have been extensively used and approved for ocular use by the FDA and EMA among others. Moreover, PHE and TPC are used as punctual mydriatic treatment and are not intended to chronic ocular administrations. Thus, the in vitro cytotoxicity on human corneal epithelial cells of the developed formulations was not superior to those of commercially available eye drops. Furthermore, corneal epithelial cytotoxicity could be reduced in regard with the lower amount of PHE and TPC that could be required to induce efficient mydriasis using in situ delivery systems. A local tolerance study in vivo should complete these results.

### 3.4. In Vivo Evaluation of the Mydriasis

**Figure 6:**
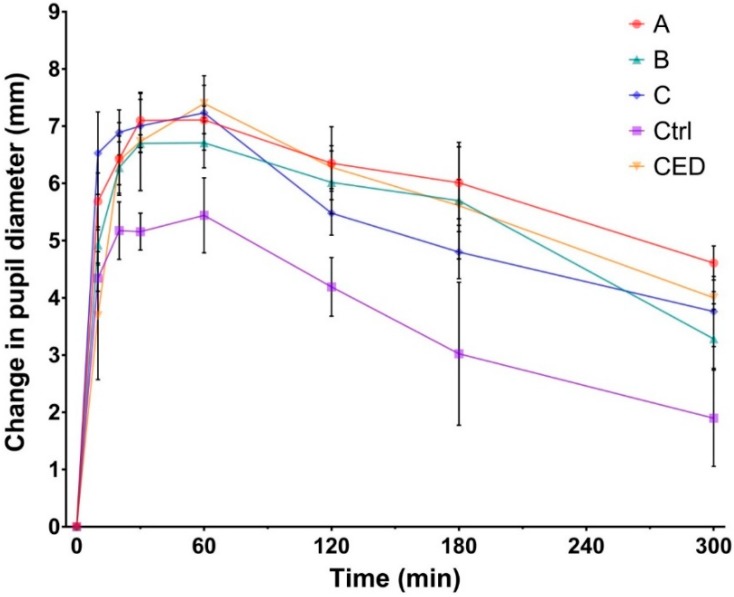
Mydriasis response profiles displayed as change in pupil diameter versus time following the administration of one drop of formulation A, B, and C compared with the administration of one drop of a liquid control solution (Ctrl; tropicamide 0.5% and phenylephrine 5%) and to the administration regimen of commercially available eye drops (CED; four drops of Mydriaticum® TPC 0.5% and four drops of Neosynephrine Faure® PHE 10%).

An effective mydriasis is defined by an increase of 5 mm or more in the pupil diameter as compared with reference diameter (t0) and the absence of pupil reflex [44]. It can be observed from Figure 6 that formulations A, B, and C allowed achieving efficient mydriasis in less than 10 min and maintained a sufficient dilation for more than 3 h. On the contrary, the control solution allowed achieving efficient mydriasis in about 20 min and maintained a sufficient effect for less than 1 hour. It is important to notice that one drop of developed formulations contained the same amount of PHE and TPC than one drop of control solution. Therefore, the same amount of PHE and TPC were instilled.

**Figure 7:**
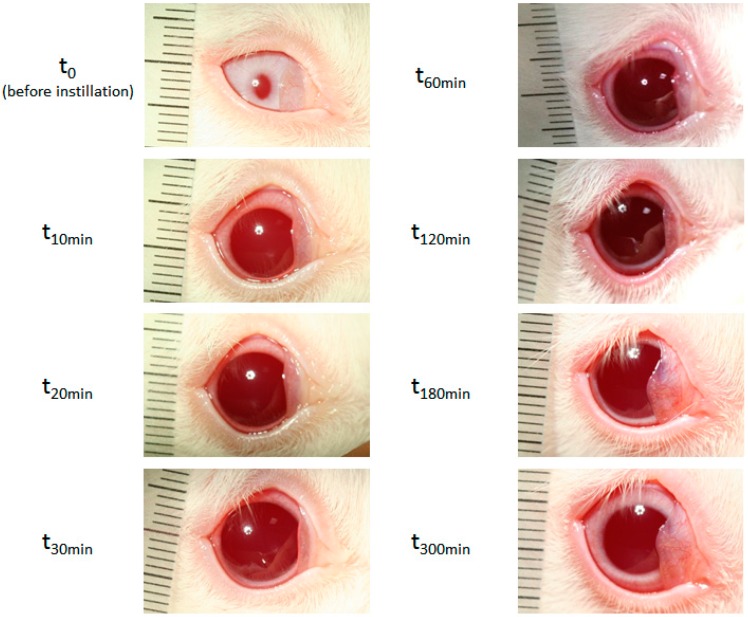
Pictures recorded 5 h following the administration of in situ gelling delivery system (Formulation A).

The pupil dilation induced by the administration of one drop of formulation A in rabbit eye is shown on Figure 7.

Area under curves were calculated and a significant difference was exhibited between formulations A, B, and C compared with the control solution, highlighting the direct effect of in situ gelling formulations on bioavailability (Table 8). This significant increase in pupil dilation could be explained by the prolonged residence time and sustained drug release previously assessed for the developed in situ gelling delivery systems.

Following the administration of commercially available eye drops (CED), efficient mydriasis was achieved in about 20 min and was maintained for 3 h. Except for the first 20 min, where the intensity of mydriasis following administration of CED was significantly lower than those of formulations A, B, and C, the mydriasis intensity profiles were then comparable (Figure 6).

All developed formulations allowed an efficient mydriasis following a single drop administration. Moreover, sufficient mydriasis was obtained faster than when using the reference protocol. The intensity and duration of mydriasis induced by formulations A, B, and C were similar to those obtained following administration of CED (Figure 6, Table 8). 

Thus, the developed in situ gelling delivery systems can be considered as promising alternatives to existing treatments. Efficient mydriasis can be induced following the instillation of a single liquid drop, which undergoes gelation upon administration on the ocular surface, leading to enhanced residence time and sustained drug delivery, increasing bioavailability drastically.

Moreover, the amount of PHE and TPC administered following the CED protocol were greater, by eight- and four-fold, respectively. Nevertheless, pupil dilation was slower to appear and did not exhibit longer duration, highlighting the lower bioavailability and higher elimination rate of the drugs from the ocular surface following administration of conventional eye drops.

Hence, the developed in situ gelling delivery systems could lead to a decrease of the risk of side effects. On the one hand, local irritations of the ocular surface region could be reduced as the total amount of active ingredients required to induced mydriasis was smaller using developed in situ gelling delivery systems compared as CED. PHE and TPC exhibited an important cytotoxicity on human corneal epithelial cells. During the experiments, discomfort resulting in increased blink frequency was observed following phenylephrine eye drop (Neosynephrine^®^ 10%) administration (five to six additional blinks within 30 seconds following administration), while neither an increase in the blink frequency or any sensation of discomfort was observed after administration of formulations A, B, and C. This effect could be due to the high osmolality (>900 mOsm/kg) of phenylephrine eye drops. A stinging sensation has been reported many times in humans after administration of this eye drops [45]. The comfort of the patient could therefore be improved using in situ gelling delivery systems. On the other hand, systemic side effects could be reduced as a result of the decrease of the drug elimination from the ocular surface potentially absorbed at the systemic level. Indeed, enhanced ocular residence time allowed retaining PHE and TPC on the ocular surface region leading to a greater amount of drug absorbed locally.

## 4. Conclusions

In this study, all in situ gelling delivery systems exhibited sustained drug release. Drug release mechanisms were studied, and diffusion was pointed out as the principle release mechanism. The addition of HEC enhanced the gels resistance to erosion at high flow rates. These findings were consistent with SEM observations of polymer network microstructures. Additionally, the developed in situ gelling delivery systems showed no cytotoxicity on human corneal epithelial cells, but the active ingredients cytotoxicity was revealed. Subsequently, the developed formulations exhibited certain cytotoxicity in vitro which was similar to those of commercially available mydriatic eye drops. Further studies on the local tolerance in vivo could allow assessing the safety of the in situ gels. Mydriasis intensity and kinetic was assessed in vivo and compared with existing mydriatic strategies.

These new formulations are promising alternatives to available mydriasis-inducing strategies regarding their capacity to induce a fast, long-lasting, and efficient mydriasis. The easy and safe administration of a single liquid drop was sufficient. The in situ gelation and sustained drug release of developed formulations allowed a significant increase in mydriasis intensity compared with liquid eye drops. Therefore, the number of instilled drops and the amount of active ingredients required to induce efficient mydriasis were reduced by four to eight-fold, drastically decreasing the risks of local toxicity and systemic side effects. Finally, these innovative mydriatic formulations could be an added value to patient management; a reduction of the medical care time prior to ocular surgeries and ophthalmic examinations is expected, making it possible to best meet patients and practitioners needs.

## Figures and Tables

**Table 1:** Composition of the in situ gelling delivery systems.

Formulation	Gellan Gum (% *w*/*v*)	Hydroxyethylcellulose (% *w*/*v*)	Sodium Citrate (% *w*/*v*)	Phenylephrine (% *w*/*v*)	Tropicamide (% *w*/*v*)
A	0.15	0	0.09	5.0	0.5
B	0.15	0.25	0.09	5.0	0.5
C	0.15	0.5	0.09	5.0	0.5

**Table 2:** Applied mathematical models and their descriptions [31].

Model	Equation	Description
Higuchi	$\frac{M_{t}}{M_{\infty }}=k_{H}t^{1/2}$	Diffusion as release mechanism
Korsmeyer-Peppas	$\frac{M_{t}}{M_{\infty }}=k_{KP}t^{n}$	0.3 < *n* < 0.5, Fickian/diffusion-controlled release; *n* = 1.0, zero-order release; 0.5 < *n* < 1.0, anomalous/co-existence of diffusion and erosion
Peppas-Sahlin	$\frac{M_{t}}{M_{\infty }}=k_{1}t^{1/2}k_{2}t$	Separation of diffusion part and erosion parts
Weibull	$\frac{M_{t}}{M_{\infty }}=1-e^{\frac{-t^{\beta }}{\alpha }}$	*β* < 0.75, Fickian/diffusion-controlled release; 0.75 < *β* < 1.0, anomalous/co-existence of diffusion and erosion
Hopfenberg	$\frac{M_{t}}{M_{\infty }}=1-{\left(1-\frac{k_{0}t}{c_{0}a}\right)}^{n}$	Erosion dependent release, not influenced by diffusion
Makoïd-Banakar	$\frac{M_{t}}{M_{\infty }}=k_{MB}t^{n}e^{\left(-ct\right)}$	*c* = 0.0, Korsmeyer-Peppas power law; If *c* ≈ 0.0, same interpretation of *n* as in Korsmeyer-Peppas model with a better fit to experimental values

*M_t_*/*M_∞_*: fraction of drug release at each time point *t*; *k_H_*: Higuchi release kinetic (diffusion) constant; *k_KP_*: kinetic constant; *n*: release exponent that is indicative of release mechanism; *k_1_*: diffusion constant; *k_2_*: relaxation (erosion) constant; *α*: scale parameter; *β*: shape parameter; *k_0_*: erosion rate constant; *c_0_*: initial concentration of drug; *k_MB_*: kinetic constant.

**Table 3:** Similarity factor f2 pairwise comparison of the drug release profiles at 3, 8, and 15 mL/min flow rates. Intra-formulations comparison of PHE and TPC release profiles, inter-formulations comparison of PHE release profiles and inter-formulations comparison of TPC release profiles.

Drug Release Profile	Formulation	f2 Values (3 mL/min)	f2 Values (8 mL/min)	f2 Values (15 mL/min)
PHE; TPC	PHE A; TPC A	60	66.8	84.4
	PHE B; TPC B	58.7	63.8	80.0
	PHE C; TPC C	57.2	63.4	68.9
PHE; PHE	PHE A; PHE B	93.9	63.2	64.1
	PHE A; PHE C	95.6	48.9	35.0
	PHE B; PHE C	93.3	72.8	39.9
TPC; TPC	TPC A; TPC B	89.7	59.9	62.7
	TPC A; TPC C	90.2	49.4	32.6
	TPC B; TPC C	96.9	72.3	37.7

**Table 4:** Similarity factor f2 pairwise comparison of phenylephrine and tropicamide release profiles. Intra-formulations comparison at different flow rates.

Flow Rates Comparisons (mL/min)	f2 Values					
	PHE A	PHE B	PHE C	TPC A	TPC B	TPC C
3 vs 8	49.0	62.9	86.1	46.5	57.6	72.2
3 vs 15	27.7	30.8	87.2	24.3	27.7	72.6
8 vs 15	36.1	35.8	89.7	32.8	33.3	79.4

**Table 5:** Goodness of fit and relevant parameters values of applied mathematical models.

Flow Rate	F	Peppas-Sahlin			Makoïd-Banakar			Weibull		Hopfenberg
		*R* ^2^_adj_	*k_1_*	*k_2_*	*R* ^2^_adj_	*n*	*C*	*R* ^2^_adj_	*β*	*R* ^2^_adj_
3 mL/min	A	0.999	10.48	0.254	0.999	0.46	0.001	0.999	0.61	0.898
	B	0.999	10.11	0.222	1.000	0.45	0.001	0.999	0.61	0.899
	C	0.999	10.59	0.280	0.999	0.47	0.002	0.999	0.59	0.889
8 mL/min	A	0.999	12.90	0.334	0.999	0.41	0.001	0.998	0.65	0.941
	B	0.999	12.36	0.425	1.000	0.34	0.002	0.998	0.56	0.893
	C	0.999	10.66	0.235	1.000	0.39	0.002	0.998	0.61	0.898
15 mL/min	A	0.984	13.03	0.63	0.999	0.85	0.019	0.999	0.95	0.998
	B	0.978	9.99	1.02	0.998	1.01	0.025	0.997	0.99	0.998
	C	0.996	10.56	0.22	0.998	0.58	0.008	0.998	0.58	0.793

**Table 6:** T_25_, T_50_, and T_80_ of formulations A, B, and C.

Flow Rate	Formulation	T_25_ (min)		T_50_ (min)		T_80_ (min)	
		PHE	TPC	PHE	TPC	PHE	TPC
3 mL/min	A	6.5	10.4	30.3	42.3	102.1	110.6
	B	6.9	10.9	31.9	45.3	103.9	121.4
	C	6.4	10.6	30.6	45.2	108.6	125.3
8 mL/min	A	4.2	5.6	19.1	24.2	60.2	68.8
	B	4.8	6.4	23.6	31.7	94.7	125.7
	C	6.2	8.4	28.2	37.4	89.9	113.5
15 mL/min	A	3.1	3.5	10.9	11.8	24.5	25.5
	B	4.3	4.9	13.3	14.4	27.2	28.7
	C	6.2	8.6	28.4	34.7	89.2	108.9

**Table 7:** Drug release kinetic parameters of formulations A, B, and C in a semi-solid adapter.

Formulation		T_25_ (h)	T_50_ (h)	T_80_ (h)
PHE	A	0.8	3.1	7.9
	B	0.9	3.6	9.2
	C	1.0	4.2	10.6
	Ctrl	0.3	1.5	3.7
TPC	A	0.9	3.9	10.0
	B	1.3	5.1	13.1
	C	1.5	6.1	15.6
	Ctrl	0.4	1.6	3.9

**Table 8:** Area under ‘the change in pupil diameter versus time’ curve (AUC_0__–300 min_).

Formulation	AUC_0__–__300 min_ (mm min)
A	1781.6 ± 121.7
B	1618.8 ± 151.8
C	1586.2 ± 120.9
CTRL	1080.5 ± 169.1
CED	1690.6 ± 97.6
